# Inhibition of C3 Convertase Activity by Hepatitis C Virus as an Additional Lesion in the Regulation of Complement Components

**DOI:** 10.1371/journal.pone.0101422

**Published:** 2014-07-01

**Authors:** Hangeun Kim, Keith Meyer, Adrian M. Di Bisceglie, Ranjit Ray

**Affiliations:** 1 Department of Internal Medicine, Saint Louis University, St. Louis, Missouri, United States of America; 2 Department of Molecular Microbiology & Immunology, Saint Louis University, St. Louis, Missouri, United States of America; Rutgers, The State University of New Jersey, United States of America

## Abstract

We have previously reported that *in vitro* HCV infection of cells of hepatocyte origin attenuates complement system at multiple steps, and attenuation also occurs in chronically HCV infected liver, irrespective of the disease stage. However, none of these regulations alone completely impaired complement pathways. Modulation of the upstream proteins involved in proteolytic processing of the complement cascade prior to convertase formation is critical in promoting the function of the complement system in response to infection. Here, we examined the regulation of C2 complement expression in hepatoma cells infected *in vitro* with cell culture grown virus, and validated our observations using randomly selected chronically HCV infected patient liver biopsy specimens. C2 mRNA expression was significantly inhibited, and classical C3 convertase (C4b2a) decreased. In separate experiments for C3 convertase function, C3b deposition onto bacterial membrane was reduced using HCV infected patient sera as compared to uninfected control, suggesting impaired C3 convertase. Further, iC3b level, a proteolytically inactive form of C3b, was lower in HCV infected patient sera, reflecting impairment of both C3 convertase and Factor I activity. The expression level of Factor I was significantly reduced in HCV infected liver biopsy specimens, while Factor H level remained unchanged or enhanced. Together, these results suggested that inhibition of C3 convertase activity is an additional cumulative effect for attenuation of complement system adopted by HCV for weakening innate immune response.

## Introduction

A significant number of people infected with HCV develop chronic infection [1], [2]. Hepatocytes are the primary host for HCV replication and serve as a main source for complement synthesis. We previously examined the relationship between HCV infection and complement regulation, and have shown that HCV infection attenuates complement system by modulating multiple components, such as C3, C4, and C9 [3]–[5]. The complement system plays a central role in the innate immune system, as a first line of defense against pathogen infection. The complement system quickly detects antibody bound microbes for elimination. All three complement activation pathways (classical, lectin, and alternative), merge for the cleavage of C3 in to C3a and C3b by C3 convertase. Cleavage of C3 by C3 convertases results in the formation of C3b and the anaphylatoxin C3a. Further processing of C3b results in the formation of iC3b and C3f, and finally C3c and C3dg [6]. In this process, Factor I is a key serine protease that inactivates all complement pathways by degrading activated complement factors C4b and C3b. Factor I degrades C4b and C3b only in the presence of specific cofactors, such as Factor H, C4b binding protein (C4BP), membrane-cofactor protein (MCP), and complement receptor 1 (CR1) [7]. Deficiencies in complement predispose patients to infection via ineffective opsonization, and defects in membrane attack complex (MAC) mediated lysis activity [8], [9]. Therefore, insights into the mechanisms of complement regulation are crucial for understanding disease pathology and therapies.

Complement component 2 (C2) is a 110 kDa serum glycoprotein that functions as part of the classical pathway of the complement system. The key function of C2 is the formation of the classical C3 convertase (C4b2a) together with C4b [8]. C2 deficiency (C2D) is the most common of the complement component deficiency. Hereditary C2D is an important susceptibility factor for invasive infections caused by encapsulated bacteria, such as pneumococci and haemophilus influenza type b [10]–[16]. C2D may also be a risk factor for development of atherosclerosis. However, many persons with C2D are apparently healthy. Complement component 3 (C3) plays an essential role in the complement pathways, including mediating convertase activity, opsonization, anaphylotoxin production, B cell activation, immunoglobulin production, immune-complex clearance. C2 is one of the C3 convertase components. C3 deficiency, either genetically determined or caused by deficiencies in the regulatory proteins factor H or factor I, include increased susceptibility to infection and rheumatic disorders [16], [17]. In this study, we examined the effect of HCV upon C2 at the transcriptional level in HCV infected patient liver biopsies and in infected patient sera on the formation and activation of C3 convertase.

## Materials and Methods

### Reagents

Mouse monoclonal antibody to human C3 (Abcam, MA), goat anti-mouse secondary antibody (Sigma, MO), purified human complement component C3 protein (Quidel, CA) were purchased.

### Patient materials

Paired serum samples and liver biopsy specimens from 12 chronically HCV infected patients [3], [4] and 12 non-HCV liver disease patients were randomly selected for use in this study. Sera and liver samples were collected from subjects with their written consent, and the protocol was approved by the Saint Louis University Internal Review Board. The liver biopsy specimens were read by an experienced pathologist, and the severity of hepatitis was graded and staged according to a system described by Scheuer [18]. Liver biopsy tissue specimens were embedded in optimum cutting temperature (OCT) formulation and preserved at −70°C. Sera were prepared after storing on ice within a short time (2–3 h) to retain complement activity, aliquoted, and stored at −70°C. Each aliquot was thawed and tested once for complement related activity. Commercially available control liver RNAs were procured (Clonetics, CA; Clonetech, CA; and Lonza, NJ).

### Cell culture grown HCV and infection of cells

Immortalized human hepatocytes (IHH) or human hepatoma cells were used to grow HCV genotype 1a, as previously described [19]–[20]. Cell culture grown HCV released in culture supernatant was filtered through a 0.45 µm cellulose acetate membrane (Nalgene, NY), aliquoted, and stored at −70°C for single use. RNA (IU/ml) was quantified by real-time PCR (Prism7500 real-time thermocycler; ABI) with the use of HCV analyte-specific reagents (ASR, Abbott) in the Pathology Clinical Laboratory at Saint Louis University. Infectious virus titer from cell culture supernatant was also measured by fluorescence-focus forming unit (FFU) using a NS5A specific monoclonal antibody. For virus infection, hepatocytes were incubated with 0.5 moi of cell culture grown HCV. After virus adsorption for ∼8 h, cells were washed, incubated with fresh medium for 4 days, and used in subsequent experiment.

### ELISA

HCV infected patient sera were used for C3 convertase (USCNK, TX) and iC3b (Quidel, CA) analyses by a commercially available enzyme-linked immunosorbent assay (ELISA) kit following manufacturer's protocol.

### C3 cleavage assay

50 µg/ml of C3 purified protein was incubated with an indicated dose of human sera at 37°C for 30 min. Samples were separated under reducing conditions by SDS-PAGE. Proteins were transferred onto nitrocellulose membrane and treated with anti-human C3 antibody at 4°C overnight, followed by a mouse secondary antibody. The experiment was carried out three times, and a representative figure is shown. Densitometry was analyzed using Image J software.

### C3b deposition assay

C3b deposition on gram-negative bacterial cell surface was performed similarly as described [21]. Briefly, *E. coli* DH5α (∼1×10^7^) was incubated with HCV infected patient serum or normal human serum (NHS) (10% sera in a final volume of 50 µl) at 37°C for 30 min. Purified C3 protein (20 µg/ml) was added in the bacteria and serum mixture. After washing with PBS, opsonized log-phase bacteria were plated onto 96-well EIA plates at ∼5×10^6^ per well and adhered by dry desiccation. Nonspecific binding sites were blocked with 0.5% BSA in PBS and incubated for 30 min at 37°C. After washing, the plates were sequentially incubated at room temperature with C3 antibody for 60 min, HRP-conjugated secondary antibody for 30 min, and finally substrate solution for visualizing HRP activity. The reaction was stopped with H_2_SO_4_ and color intensity measured at OD_490_.

### Convertase functional assay

Amboceptor sensitized sheep erythrocytes (EA) were purchased (Complement Technologies, Tyler, TX). Erythrocytes (5×10^8^) were washed with dextrose gelatin veronal buffer (DGVB) buffer (2.5 mM veronal buffer, pH 7.3, 72 mM NaCl, 140 mM glucose, 0.1% gelatin, 1 mM MgCl_2_, and 0.15 mM CaCl_2_), EA suspensions were adjusted and lysed with 90 µl of water to have an absorbance value of ∼1.5 at 405 nm.

For assessment of convertase function [22], 10 µl of EA were placed in a 96-well V-shape microplate (Nunc) and 40 µl of DGVB buffer containing a 2.0% HCV patient or healthy control serum was added to assemble convertases. The plate was kept in an incubator shaker at 30°C with 300 rpm shaking for 5 minutes for convertase formation. To initiate complement mediated lysis from existing convertase complexes, 50 µl of 40 mM EDTA-GVB buffer (40 mM EDTA, 5 mM veronal buffer, 0.1% gelatin, 145 mM NaCl) containing 1∶40 guinea pig serum as a source of complement was added and the plate was incubated for another 30 minutes at 37°C on a 300 rpm shaker. Alternatively, lysis was developed with guinea pig complement deficient in C4 in 40 mM EDTA-GVB buffer. Cells were pelleted and 50 µl of the supernatant containing hemoglobin released from lysed EA were measured at 405 nm in a microplate reader.

### Blocking of Factor B activity in a classical C5 convertase assay

To assess the contribution of the alternative convertases as an amplification loop of the classical pathway, we blocked Factor B activity during and after convertase formation. EA were mixed with 40 µl DGVB containing serum at a final concentration of 2% and 60 µg/ml of function blocking mouse anti-human FB antibody (Quidel, SanDiego) or control antibody. C5 convertase was allowed to assemble as previously described [22]. To initiate complement-mediated lysis, the same dilution of anti-Factor B antibody in 50 µl of DGVB was added and incubated in the presence of a 1∶40 dilution of guinea pig complement. Cells were pelleted and supernatant containing hemoglobin released was measured at 405 nm.

### Real-time PCR

C2, Factor I, or Factor H mRNA quantitation was performed by real-time PCR using specific primers and probes using a 7500 Fast Real Time PCR system (Applied Biosystems, CA). RNA was isolated from experimental samples using TRIzol (Invitrogen, CA), and cDNA was synthesized using random hexamers and a SuperScript III first-strand synthesis kit (Invitrogen).

### Statistical analysis

Results were expressed as the mean ± standard deviation (SD), and statistical analyses were performed using a two-tailed unpaired Student *t* test or one-way analysis of variance (ANOVA) in GraphPad Prism, version 5 (GraphPad, La Jolla, CA). A p values <0.05 was considered statistically significant.

## Results

### HCV infection inhibits C2 mRNA expression

**Figure 1:**
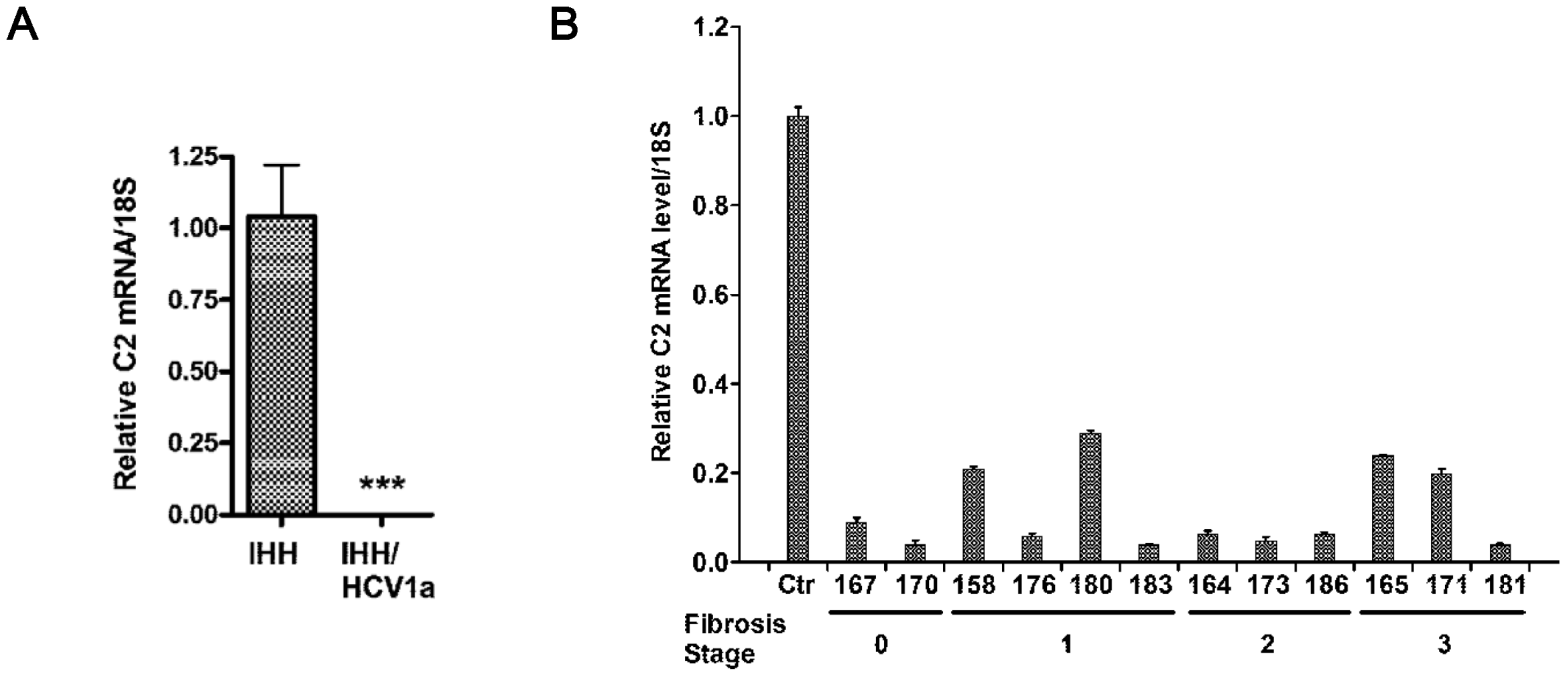
HCV infection inhibits mRNA expression of C2 complement component. (A) Real-time PCR analysis for C2 mRNA expression from HCV infected IHH and mock infected control cells. Results were normalized to endogenous 18S RNA. Asterisks denote: *p<0.05; **p<0.01; ***p<0.001 when compared to controls. (B) Real-time PCR analysis displaying HCV genotype 1a infection represses C2 expression in chronically infected liver biopsy specimens from patients (n = 12). Liver biopsy samples are each marked by 3-digit numbers at the bottom. Results are compared with non-HCV infected 3 control liver tissues arbitrarily set at 1. The error bars in individual samples are shown as variations form triplicate assays. All samples used in this study were significantly inhibited (p<0.01) as compared to negative controls.

We analyzed C2 mRNA expression in cells infected with cell culture-grown HCV. A significant reduction of C2 mRNA expression was observed in HCV genotype 1a infected IHH, as compared to mock-infected controls (Fig. 1, panel A). The results clearly indicated that HCV infection regulates C2 complement component at the transcriptional level at different magnitude based on HCV genotype.

In order to investigate the effect of HCV infection on C2 expression in chronically infected patients, C2 mRNA status was measured from liver biopsy samples. For this, RNA was extracted from randomly selected 12 liver biopsy patients infected with HCV genotype 1a [4], [5]. Three unrelated human liver RNAs were included in this study for comparison. C2 mRNA expression was measured by real-time PCR. All HCV-infected liver biopsy specimens exhibited a significant reduction in C2 mRNA expression (>70%) as compared to control human liver RNAs (Fig. 1, panel B). Our results exhibited C2 repression in 12 liver biopsy specimens from chronically HCV infected patients. The C2 expression status was independent of their fibrotic stage, grade, presence, or absence of rheumatoid factor.

### HCV infected patient sera exhibit reduced C3 convertase formation

**Figure 2:**
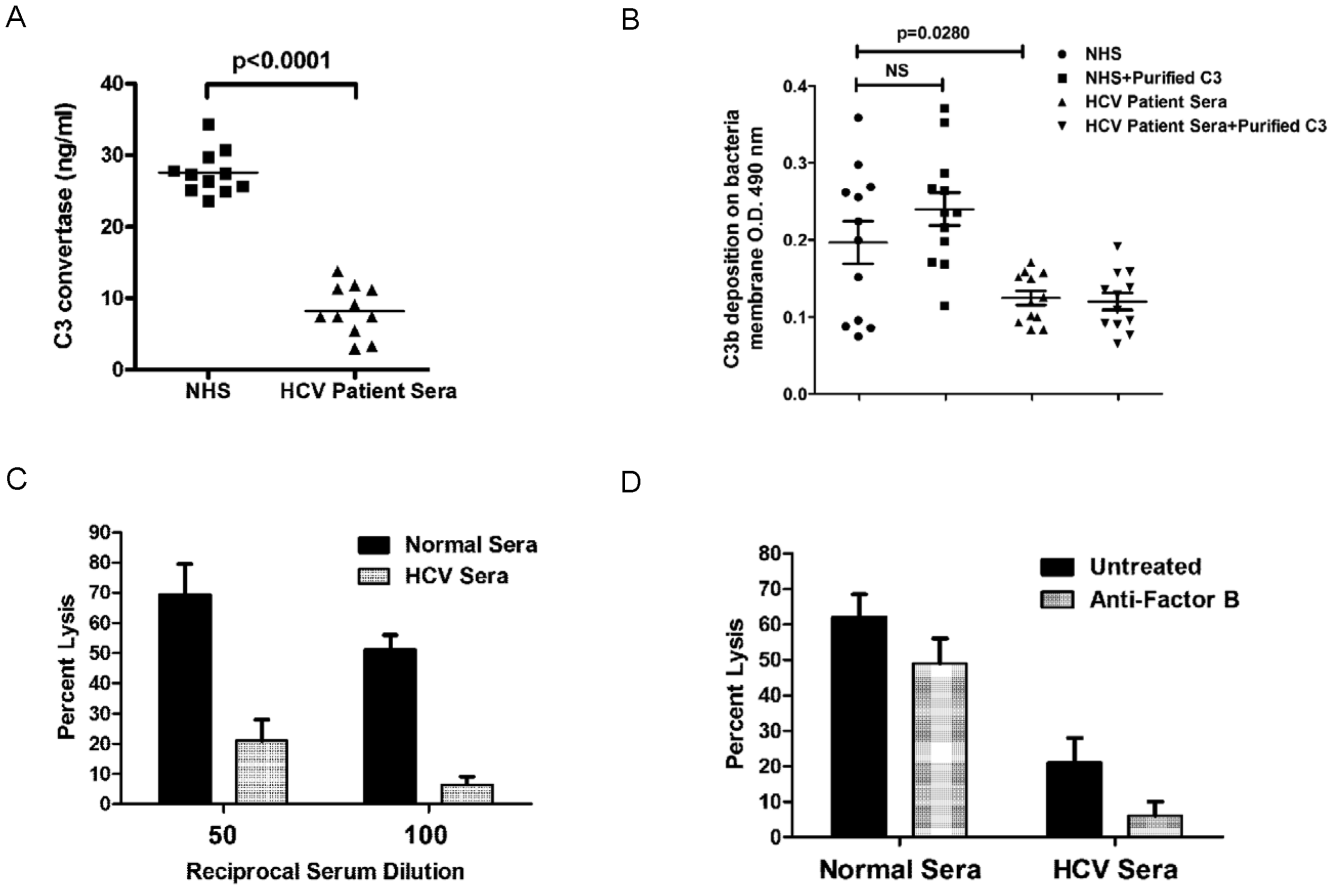
C3 convertase level reduces in HCV infected patient sera. (A) Comparison of serum C3 convertase level in randomly chosen HCV infected patient sera (n = 12) and normal human sera (NHS) from healthy donors (n = 12) using a commercially available ELISA kit. (B) HCV infected patient sera (n = 12) and NHS (n = 12) were subjected to analysis for comparison of C3b deposition on *E. coli* surface in the presence or absence of purified C3 protein. Asterisks denote: *p<0.05; **p<0.01; ***p<0.001 when compared to control. (C) Complement mediated lysis was inhibited in HCV infected patient sera. Normal sera (n = 6) and HCV infected patient sera (n = 6) were incubated with sheep RBC after treatment with EDTA. Sera from each test group were studied, with lysis of erythrocytes completed using EDTA treated guinea pig serum. (D) Sheep RBCs were incubated with sera in the presence or absence of anti-factor B antibody to separate out alternate pathway mediated lysis from classical antibody mediated lysis, and lysis was carried out as described above.

The C3 convertase status of classical pathway in normal human sera (NHS) and HCV infected patient sera was examined by ELISA. A significant inhibition of C3 convertase complex in HCV infected patient sera as compared to NHS was observed (Fig. 2, panel A). We have previously shown that C4 serum level was lower in HCV infected patients as compared to normal human sera [3]. Together, our data suggests that the reduction of complement components C2 (Fig. 1) and C4 [3], may have a direct role on C3 convertase activity of classical pathway.

We examined the C3 convertase assembly of alternative pathway by analyzing C3b deposition on bacterial surface. After cleavage of C3, C3b is deposited on pathogen surface as an initial step in the formation of C3 convertase of alternative pathway or as part of the subsequent C5 convertase [23]–[24]. We performed a C3b deposition assay using *E. coli* DH5α. The level of C3b deposition on bacteria membrane was higher in NHS as compared to HCV infected patient sera (Fig. 2, panel B), indicating a low level of C3 convertase function in HCV infected patients. The limited C3 convertase formation from both pathways and impairment of C3b generation may additionally affect C5 convertase formation in HCV infected patients. When purified C3 protein was added to the reaction, NHS had a significantly increased C3b deposition, indicating the further potential for convertase activity. However, an increase in C3b deposition was not observed in the reaction using HCV infected patient sera with the addition of purified C3 protein (Fig. 2, panel B). This result suggests that HCV infected patient sera has limited factors related to C3 processing, and convertase function is limited due to the cumulative reduction of complement components at the initial stage of convertase formation, limiting downstream protease function. C3b deposition is a critical step for the complement cascade, especially in the formation of C5 convertase, which leads to membrane attack complex (MAC) formation. Thus, impairment of C3b deposition in HCV infected patient sera can affect MAC formation and pathogen killing activity.

Further examination revealed that a panel of chronically HCV infected patient sera display significantly reduced ability to lyse antibody sensitized sheep RBC as compared to NHS as control (Fig. 2, panel C). Initial convertase formation occurred using patient and control sera, with cell lysis from added guineapig complement treated with EDTA. This protocol allows for the analysis of upstream complement dysfunction, removing the effect prior noted with the modulation of complement C9 expression [5]. Lysis of amboreceptor sensitized sheep erythrocytes was significantly reduced when HCV patient sera were used to form initial convertase function. A neutralizing antibody to Factor B was utilized to inhibit RBC lysis derived from activity of the alternate pathway (2% serum concentration), and revealed that the majority of complement activity in these antibody sensitized erythrocytes occurred through the classical (antibody mediated) pathway (Fig. 2, panel D) in NHS. A similar reduction of alternate pathway mediated lysis was also apparent in HCV infected patient sera with the use of Factor B antibody. In the final analysis, the data indicate that the classical pathway mediated lysis analyzed in this system was compromised by a nearly 10 fold reduction in activity of HCV patient sera as compared to NHS. These results indicate that the expression and proteolytic processing of the early components of the complement convertase are significantly inhibited in HCV infected patients, with the potential to impair normal immune functions.

### Factor H and Factor I are modulated in HCV infected patient sera

**Figure 3:**
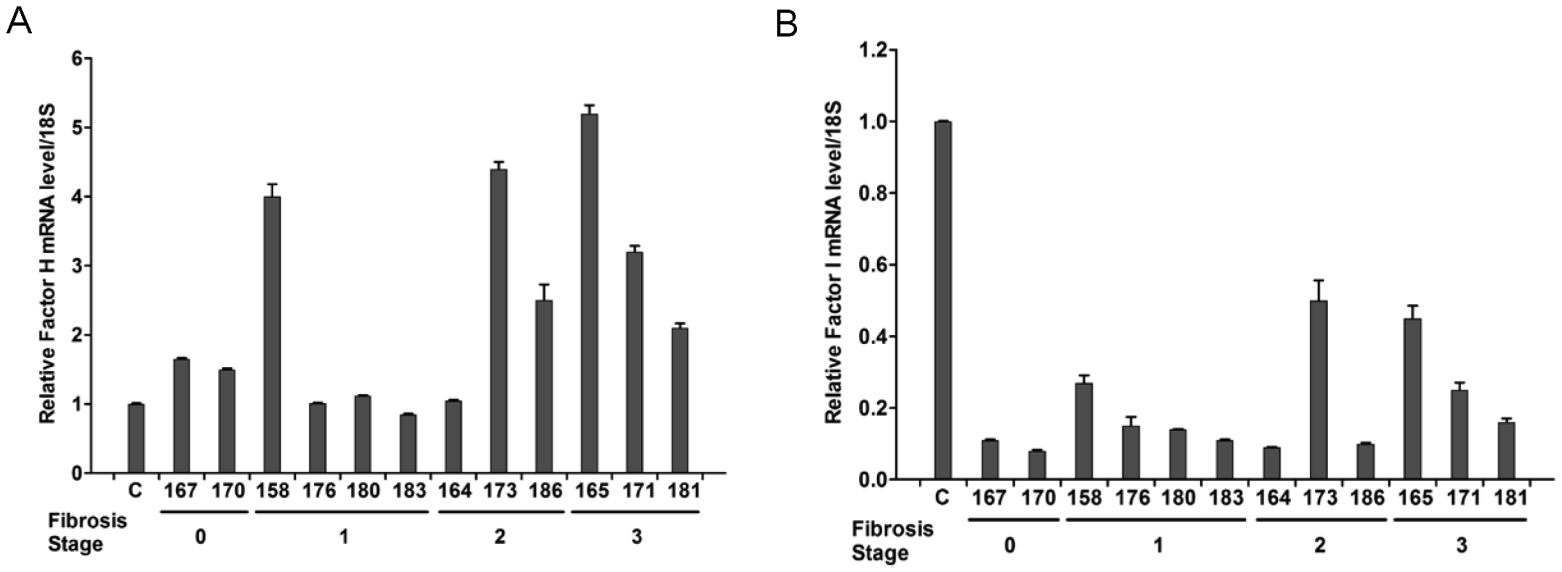
Factor H and Factor I expression are impaired in HCV infected patient sera. Real-time PCR analysis from HCV genotype 1a infected liver biopsy specimens from patients (n = 12) exhibiting Factor H (A) and Factor I (B) expression status are shown. Results were compared with non-HCV infected control liver RNAs (n = 3) arbitrarily set at 1. The error bars in individual samples are shown as variations form triplicate assays. Increase of Factor H was significant (p<0.05 in #186, 171, and 181; p<0.01 in #158, 173, and 165) as compared to control liver. Factor I inhibition was significant (p<0.05) in all tested samples.

Factor H regulates complement activation on self-cells and surfaces by possessing both cofactor activity for the Factor I mediated C3b cleavage, and decay accelerating activity against the alternative pathway C3-convertase, C3bBb [25]. Factor I is a key serine protease that inactivates all complement pathways by degrading activated complement factors C4b and C3b [7]. In this study, we examined whether HCV modulates Factor H and Factor I to influence C3 convertase. Real-time PCR analysis suggested enhanced Factor H mRNA in liver biopsy specimens of patients infected with HCV genotype 1a (Fig. 3, panel A). Six out of 12 samples displayed a significant increase in Factor H mRNA level and others had modest alterations as compared to unrelated control liver specimens, and these changes did not correlate with fibrosis stage. On the other hand, Factor I levels in liver biopsy specimens were significantly inhibited in all examined samples irrespective of liver fibrosis stage, as compared to control (Fig. 3, panel B).

### Decreased iC3b level in HCV infected patient sera

**Figure 4:**
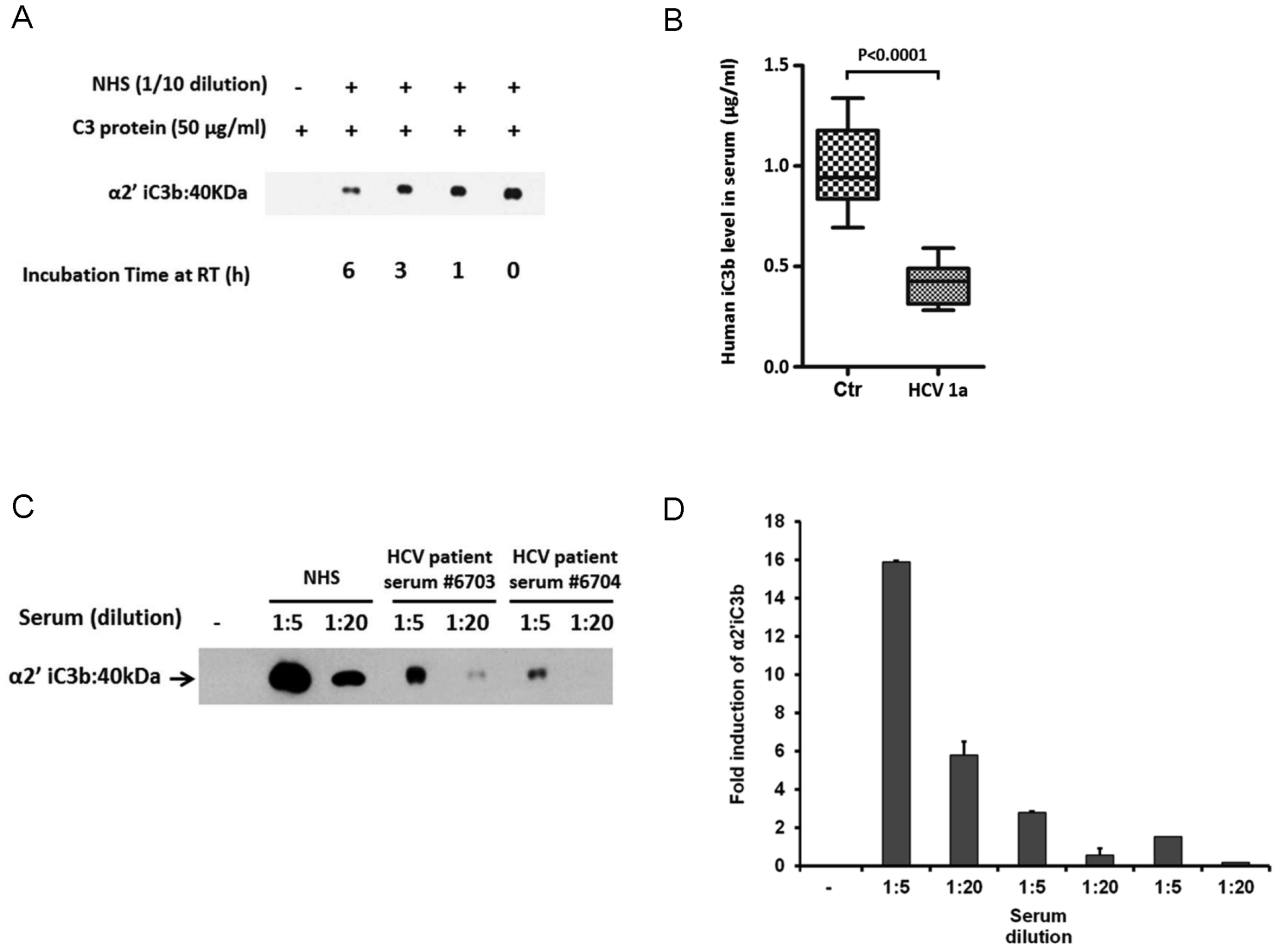
iC3b level decreases in HCV infected patient sera. (A) Undiluted NHS were incubated at room temperature for 1, 3, and 6 h. Purified C3 protein (50 µg/ml) was added to preincubated serum at 1∶10 dilution at 37°C for 30 min. The α2'iC3b protein band was detected in samples by Western blot analysis. (B) Comparison of serum iC3b level in NHS (n = 12) and HCV infected patient sera (n = 12) using a commercially available ELISA kit. (C) NHS and HCV infected patient sera incubated with purified C3 protein (50 µg/ml) at 1∶5 and 1∶20 dilution. The protein band representing α2'iC3b was detected by Western blot and a representative figure is shown. (D) Densitometry analysis for α2'iC3b protein band intensity was done by ImageJ software after repeated experiments with NHS (n = 6) and HCV infected sera (n = 6).

iC3b is generated by Factor I after formation of C3b. iC3b is a proteolytically inactive form of C3b that retains the ability to opsonize microbes, but cannot participate in convertase function. To examine the effect of time and temperature for C3 convertase, we incubated NHS at room temperature for various time and confirmed its C3 cleavage activity by observing iC3b generation. C3 convertase activity was not significantly altered by room temperature incubation up to 3 h (Fig. 4, panel A). Next, we evaluated iC3b levels in 12 chronically HCV genotype 1a infected patient sera by ELISA as an indicator of total complement activation, as C3b has a very short half-life. Six normal human sera were included as controls for comparison. The results indicated that iC3b levels were decreased at least 3 fold in HCV infected patient sera as compared to control (Fig. 4, panel B). This result suggests that C3 convertase activity was decreased in HCV infected patients. Serum iC3b level was much higher in NHS as compared to HCV infected patient sera incubated with a set concentration of purified C3 protein at the same dilution, such as 1∶5 and 1∶20 (Fig. 4, panel C). The iC3b level from 6 NHS and 6 HCV infected patient sera are represented as a fold induction after scanning of band intensity using ImageJ software (Fig. 4, panel D). These results indicated that Factor I activity is higher in NHS than in HCV infected patient sera. As we observed, Factor I quantity and/or activity is impaired in HCV infected patients, suggesting that the alternative pathway of complement leads to continuous generation of fluid-phase and cell-surface-deposited C3b by a self-amplification loop. However, C3b deposition on bacteria cell surface was limited (Fig. 2, panel B) and total C3 level was also lower in HCV infected patient sera as compared to NHS [4].

## Discussion

**Figure 5:**
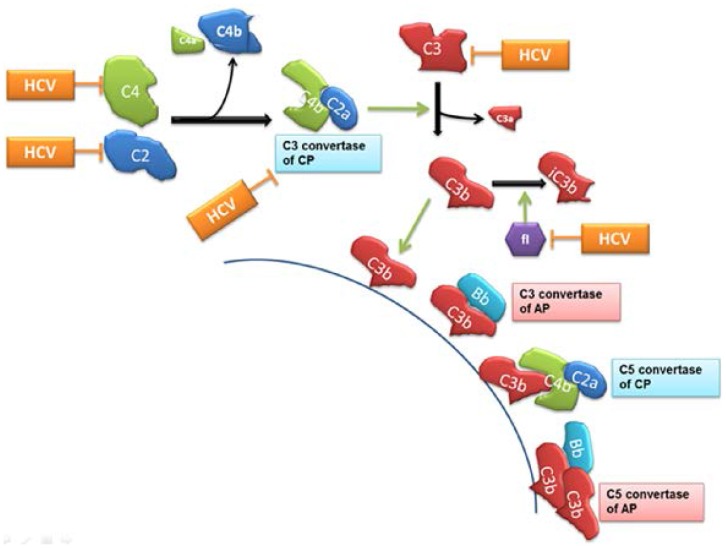
Schematic presentation of complement regulation by HCV. HCV infection inhibits C4 and C3 components expression [3], [4] as well as C2 production (current study). Reduction of C4 and C2 synthesis by HCV affects C3 convertase formation in classical pathway, which results in the inhibition of C3 cleavage. Limited C3b generation affect C3b deposition onto pathogenic surface, and impairs another C3 convertase formation of alternative pathway and C5 convertase formation in both pathways. Together with the reduction of membrane attack complex formation [5], the impairment of C3 convertase activity may be a key event for HCV persistence. CP = classical pathway, AP = alternate pathway.

The complement system plays a central role in the innate and adaptive immune response. The production of anaphylotoxins during complement convertase function results in complement induced T cell activation, with APC activation driving T cell differentiation, expansion and survival [25]. In the current study, we have shown impairment of C3 convertase in HCV infected patient sera. The effect of lower C3 convertase in the complement system leads to the impairment of downstream events including C5 convertase formation (Fig. 5). Previously, we have shown that HCV genotype 1a infected patients had a low level of C5b-9 in their sera, which resulted in impairment of MAC formation and bactericidal/virocidal activity [5]. Limited C3 convertase activity in HCV infected patients may affect C5 convertase generation and C5 cleavage. In the current study, we have shown C3b level was lower in HCV infected patient sera, suggesting that the formation of C5 convertase in classical pathway (C4b2a3b) is impaired. In addition, HCV inhibits C5 mRNA synthesis in hepatoma cells. These observations reinforce our previous results suggesting HCV impairs at multiple steps of the complement system to escape from complement mediated virocidal activity.

Factor I is a key serine protease that inactivates all complement pathways by degrading activated complement factors C4b and C3b. Factor I does not cleave intact C4 or C3, and degrades C4b and C3b only in the presence of specific cofactors, such as Factor H, C4b binding protein (C4BP), membrane-cofactor protein (MCP), and complement receptor 1 (CR1) [7]. Interestingly, C4 has been shown to a cellular substrate of HCV NS3/4A protease and may also contribute to HCV persistence [26] The physiological importance of Factor I is shown by the fact that patients lacking Factor I have increased susceptibility to recurrent infection with encapsulated microorganisms, glomerulonephritis, and rheumatologic diseases [27], [28]. In the absence of regulation by Factor I, the alternative pathway of complement leads to continuous generation of fluid-phase and cell-surface-deposited C3b by a self-amplification loop. Thus, lower level of iC3b from the current study suggests: (i) C3 cleavage occurs less in HCV infected patients because of limited C3 convertase activity, and (ii) increased iC3b level in NHS indicates the presence of activated factor I, while HCV infected patient sera has limited Factor I. Therefore, further knowledge on the mechanism of inhibition of Factor I expression by HCV may provide important information in understanding deregulation of complement activation. Factor H is a bifunctional and essential molecule for control of the alternative pathway of complement. Normal recognition of cell surface is required for effective protection. On the other hand, mutations and polymorphisms in Factor H gene may result in inadequate protection and disease. Abnormal recognition of host cell surfaces by Factor H has been associated with complement-mediated tissue damage and disease. In addition, unwanted recognition of pathogens and cancer cells by Factor H can be used as an immune evasion strategy [29]. At least 50% of HCV infected patients showed abnormal increase of Factor H and this suggest that HCV may use factor H to help escape immune surveillance, but more detailed studies are required.

Our current study suggests that HCV infection affects C3 convertase formation by modulating specific complement components and their activity. This results in an impairment of C3 processing and activation of complement dependent functions. In this manner, the effect HCV has on the innate immune system may carry over into further modulating host adaptive immune responses.
